# Assessment of the Microhardness of Three Different Glass Ionomer Cements After Microhydroxyapatite Incorporation: An In Vitro Study

**DOI:** 10.7759/cureus.71961

**Published:** 2024-10-20

**Authors:** Krishna Maity, Aishwarya Arya, Divya Mishra, Jayant Verma, Kanduri Venkata Naga Vamseekrishna, Bisma Jahangeer

**Affiliations:** 1 Conservative Dentistry and Endodontics, Private Practice, Howrah, IND; 2 Conservative Dentistry and Endodontics, Awadh Dental College and Hospital, Jamshedpur, IND; 3 Conservative Dentistry and Endodontics, Heritage Hospitals, Varanasi, IND; 4 Conservative Dentistry and Endodontics, Geetanjali Dental and Research Institute, Udaipur, IND; 5 Conservative Dentistry and Endodontics, Care Dental College, Guntur, IND; 6 Conservative Dentistry and Endodontics, Private Practice, Kulgam, IND

**Keywords:** microhardness, microhydroxyapatite, resin-modified glass ionomer, tooth structure, zirconia-reinforced glass ionomer

## Abstract

Aim

The study aimed to evaluate the impact of incorporating microhydroxyapatite on the microhardness of three different types of glass ionomer cement.

Methodology

A total of 120 disc-shaped specimens (7 mm in diameter and 3 mm in height) were prepared for the study, divided into three groups, each containing four subgroups of 10 samples. The materials tested included Zirconomer, conventional glass ionomer cement (CGIC), and resin-modified glass ionomer cement (RMGIC), with the addition of microhydroxyapatite in varying concentrations of 0, 5, 15, and 25 wt%. All specimens were stored in deionized water at 37ºC for 24 hours before being subjected to the Vickers microhardness test. Statistical analysis was performed using one-way ANOVA and Tukey's post hoc honest significance test (HSD) test, with a significance level set at p<0.05.

Results

The addition of 5 and 15 wt% microhydroxyapatite to Zirconomer and RMGIC significantly increased microhardness (p<0.001). The highest Vickers hardness number (VHN) was observed in the RMGIC group with 5 wt% of microhydroxyapatite. However, the RMGIC group with 25 wt% microhydroxyapatite showed a lower VHN compared to the control group (without hydroxyapatite) (p<0.001).

Conclusion

The incorporation of 5 and 15 wt% microhydroxyapatite enhanced the microhardness of CGIC, RMGIC, and Zirconomer. However, adding 25 wt% of microhydroxyapatite to RMGIC resulted in a reduction in hardness compared to the control group without microhydroxyapatite.

## Introduction

Dental caries has long been a significant contributor to the global burden of oral disease, leading to an ongoing quest for the ideal restorative material. In recent years, cosmetic and esthetic dentistry have become integral components of dental practice. Among the various restorative options, composite resin is widely favored by dental practitioners due to its exceptional esthetic properties and favorable handling characteristics. However, despite its excellent appearance, the use of composite resin in posterior restorations is often limited by issues such as excessive wear, polymerization shrinkage, and fracture under occlusal stress [1].

Glass ionomer cement (GIC) remains a highly valued restorative material in dentistry due to its unique properties, including its ability to bond to moist tooth surfaces, absence of exothermic polymerization, and anti-cariogenic benefits through fluoride release [2]. GIC is a water-based material, consisting of a base and an acidic catalyst. The base is typically composed of calcium or strontium aluminosilicate glass, while the catalyst contains a water-soluble polymer. When mixed, the acid reacts with the solid glass, leading to a neutralization process that results in the setting of the cement [3]. Initially discovered by Wilson and Kent in 1972, the first iteration of glass ionomer (G-200) contained a high fluoride content but was unstable [3,4]. The material, formerly known as alumino-silicate polyacrylic acid (ASPA), saw its first commercial production with ASPA I by Dentsply International's De Trey, although this early formulation was slow-setting, lacked translucency, and was vulnerable to moisture during the setting process [5].

GIC continues to be utilized in clinical dentistry for its distinct advantages, including its adhesion to tooth structure, translucency, biocompatibility, and ability to release fluoride, providing anti-cariogenic effects [6,7]. Additionally, its low coefficient of thermal expansion, which is similar to that of natural tooth structure, minimizes the risk of microleakage [8]. However, GIC also has limitations, such as brittleness, inferior mechanical properties, and sensitivity to desiccation and moisture during its initial setting phase [9,10].

To address these shortcomings, resin-modified glass ionomers (RMGICs) were developed, offering improved mechanical strength and adhesive properties, particularly in posterior restorations. RMGICs replace water with a mixture of water and hydroxyethylmethacrylate (HEMA), which allows the formation of an organic matrix, making the material more resistant to early moisture exposure. Consequently, the use of varnish or other protective coatings is not required [9,10].

A novel material, Zirconomer (often referred to as "white amalgam"), has been introduced to enhance the strength of GIC-based restorations. Zirconomer incorporates zirconia as a reinforcing agent, resulting in a high-strength restorative material. The liquid component of Zirconomer consists of zirconium oxide, glass, deionized water, tartaric acid (1-10%), polyacrylic acid (20-50%), and other compounds [11]. Zirconia, a durable ceramic, has been utilized in dentistry as a core material for crowns and fixed partial dentures due to its superior strength.

Hydroxyapatite (HAP), a bioceramic made up of calcium and phosphate (Ca10(PO4)6(OH)2), is the primary inorganic component of enamel and dentin, comprising over 60% of the latter by weight [12]. Numerous studies have demonstrated that incorporating HAP into GIC enhances its mechanical properties, including diametral tensile strength, fracture toughness, bond strength, and compressive strength. This improvement is attributed to the interaction between HAP and the carboxylate groups in the polyacid structure of GIC [13,14].

Given that mechanical strength is a key factor in the clinical success of dental restorative materials, the present study aims to investigate the microhardness of conventional GIC, RMGIC, and Zirconomer following the incorporation of microhydroxyapatite.

## Materials and methods

This in vitro investigation was carried out at the Department of Conservative Dentistry and Endodontics of Maharishi Markandeshwar College of Dental Sciences and Research in Mullana, Ambala, India.

Sample preparation

One hundred and twenty disc-shaped specimens, measuring 7 mm in diameter and 3 mm in height, were made for this experimental investigation. For this study, cylindrical molds were employed. The powder and liquid of the corresponding GIC were combined for the fabrication of specimens A, B, and C using an agate spatula and mixing pad supplied by the manufacturer, following the manufacturer's instructions. The liquid was then overfilled into the mold, and until the material was set completely, a clear Mylar Strip (Samit Matrix, India) was placed on the mold's upper surface.

In the corresponding GIC powder, groups a2, a3, a4, b2, b3, b4, c2, c3, and c4 have 5, 15, and 25 wt% of microhydroxyapatite, respectively. In accordance with the manufacturer's instructions, powder and liquid were mixed to make the samples.

Study groups

**Figure 1:**
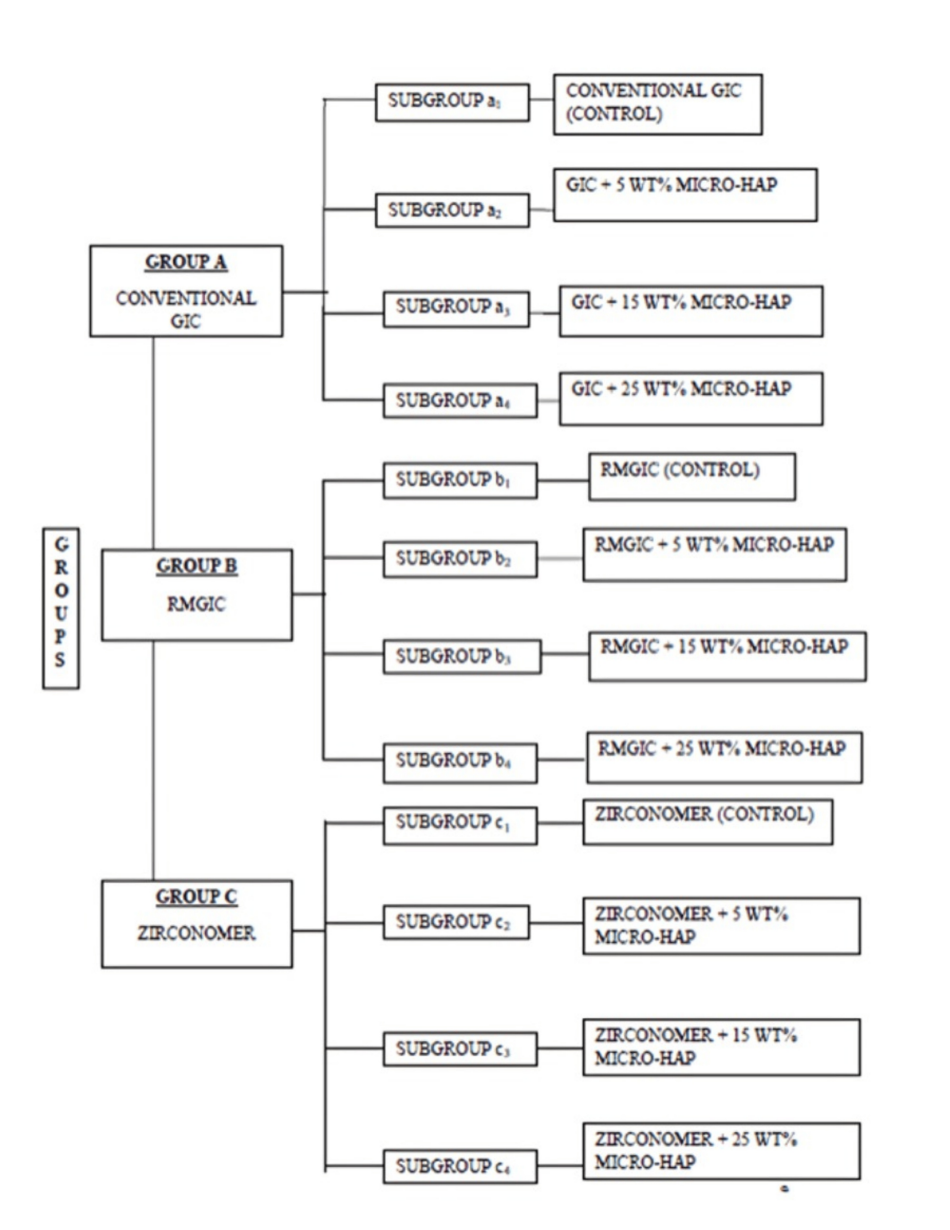
Formation of groups and study samples

The study was divided into three groups to evaluate the effects of HAP incorporation on different types of GICs. Group A focused on conventional glass ionomer cement (CGIC) and included four subgroups: a₁ (control without HAP), a₂ (5 wt% HAP), a₃ (15 wt% HAP), and a₄ (25 wt% HAP). Group B examined RMGIC with subgroups b₁ (control), b₂ (5 wt% HAP), b₃ (15 wt% HAP), and b₄ (25 wt% HAP). Group C assessed Zirconomer and was similarly divided into four subgroups: c₁ (control), c₂ (5 wt% HAP), c₃ (15 wt% HAP), and c₄ (25 wt% HAP). Each group aimed to study the effect of increasing HAP concentrations on the respective GICs' material properties (Figure 1).

Sample finishing and preservation

**Figure 2:**
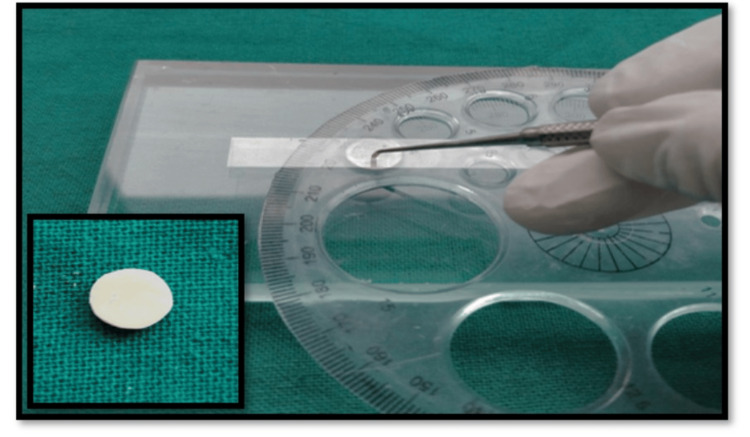
Preparation of samples and prepared samples according to groups

**Figure 3:**
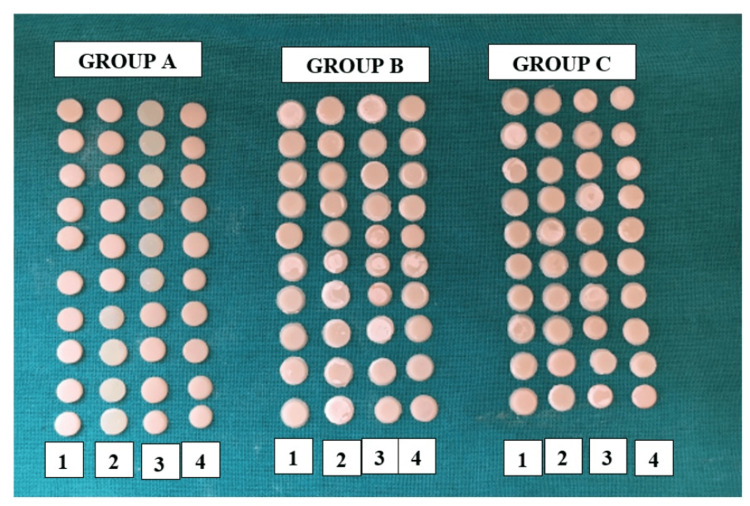
Samples of the study group

To guard against moisture, varnish was applied to every specimen. For a full day, each sample was kept in distilled water at room temperature. To polish the sample's upper and lower surfaces, Shofu Inc., Japan's Rainbow kit was used for finishing and polishing (Figure 2 and Figure 3).

Vickers microhardness measurement

**Figure 4:**
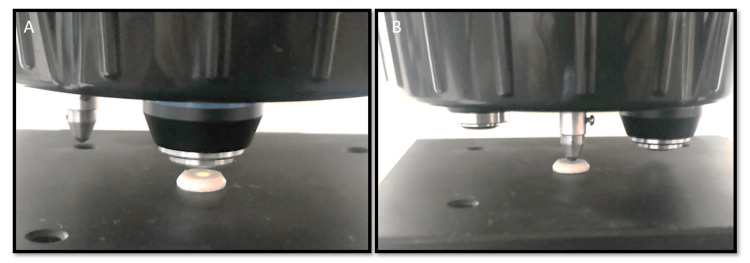
(A-B) Vickers microhardness testing in progress

The Vickers microhardness test was performed on each sample. A load of 300 gr (2.94 N) was applied during the test, which was conducted using a Vickers microhardness tester (Vaiseshika Pvt. Ltd., India) (Figure 4).

The gathered data were subjected to statistical analysis for additional review, and conclusions were drawn using statistical data. The number of cases and percentages were used to display the data. Tukey's post hoc honest significance test (HSD) test and the one-way ANOVA test were used to analyze continuous data. Using a two-tailed test, significance was determined by p-values less than 0.05. IBM SPSS Statistics for Windows, Version 21.0 (Released 2012; IBM Corp., Armonk, New York, United States) was used for data analysis.

## Results

The aim of the present investigation was to conduct a comparative analysis of the microhardness of three distinct categories of GICs (namely, CGIC, RMGIC, and Zirconomer) subsequent to the inclusion of 5 wt%, 15 wt%, and 25 wt% of microhydroxyapatite.

In the CGIC group, there was a statistically significant difference in microhardness between the experimental groups (p<0.0001) (Table 1).

**Table 1:** Comparison of microhardness between the CGIC group CGIC: conventional glass ionomer cement; HAP: hydroxyapatite

Groups	Mean	Std. deviation	P-value
CGIC (control)	44.16	6.82	<0.0001
CGIC+5 wt% HAP	79.68	6.00	
CGIC+15wt% HAP	66.06	6.99	
CGIC+25wt% HAP	51.61	6.62	

The microhardness in the 5 wt% HAP group was 79.68±6.00, and in the 15 wt% HAP group, it was 66.06±6.99. Intragroup comparisons revealed a statistically significant difference between the control group and both the 5 wt% HAP and 15 wt% HAP groups. However, no statistically significant difference was observed between the control and 25 wt% HAP groups, with a mean difference of -7.45 (p=0.099) (Table 2).

**Table 2:** Intragroup comparison between microhardness in the CGIC group (one-way ANOVA) CGIC: conventional glass ionomer cement; HAP: hydroxyapatite *The intragroup comparison shows there is a statistically significant difference between control and 5 wt% HAP and 15 wt% HAP and there is no statistically significant difference between control and 25 wt% HAP with a mean difference of -7.45 (p=0.099).

Groups		Mean difference (I-J)	P-value
CGIC (control)	CGIC+5 wt% HAP	-35.51700^*^	<0.0001
	CGIC+15 wt% HAP	-21.89600^*^	<0.0001
	CGIC+25 wt% HAP	-7.44800	0.099
CGIC+5 wt% HAP	CGIC (control)	35.51700^*^	<0.0001
	CGIC+15 wt% HAP	13.62100^*^	<0.0001
	CGIC+25 wt% HAP	28.06900^*^	<0.0001
CGIC+15 wt% HAP	CGIC (control)	21.89600^*^	<0.0001
	CGIC+5 wt% HAP	-13.62100^*^	<0.0001
	CGIC+25 wt% HAP	14.44800^*^	<0.0001
CGIC+25 wt% HAP	CGIC (control)	7.44800	0.099
	CGIC+5 wt% HAP	-28.06900^*^	<0.0001
	CGIC+15 wt% HAP	-14.44800^*^	<0.0001

For the RMGIC group, a statistically significant difference in microhardness was also found between the experimental groups (p<0.0001) (Table 3).

**Table 3:** Comparison of microhardness between the RMGIC group RMGIC: resin-modified glass ionomer cement; HAP: hydroxyapatite

Groups	Mean	Std. deviation	P-value
RMGIC (control)	55.51	5.69	<0.0001
RMGIC+5 wt% HAP	80.41	8.75	
RMGIC+15 wt% HAP	69.94	15.34	
RMGIC+25 wt% HAP	42.20	6.26	

The 5 wt% HAP group exhibited a microhardness of 80.41±8.75, while the 15 wt% HAP group showed 69.94±15.34. Intragroup comparisons indicated a statistically significant difference between the control group and the 5 wt% HAP, 15 wt% HAP, and 25 wt% HAP groups. However, no significant difference was observed between the control group and both the 5 wt% HAP and 15 wt% HAP groups (Table 4).

**Table 4:** Intragroup comparison between microhardness in the RMGIC group RMGIC: resin-modified glass ionomer cement; HAP: hydroxyapatite *The intragroup comparison shows there is a statistically significant difference between control and 5 wt% HAP, 15 wt% HAP, and 25 wt% HAP and there is no statistically significant difference between control and 5 wt% HAP and 15 wt% HAP.

Groups		Mean difference (I-J)	P-value
RMGIC (control)	RMGIC+5 wt% HAP	-24.90000^*^	<0.0001
	RMGIC+15 wt% HAP	-14.43300^*^	0.013
	RMGIC+25 wt% HAP	13.31100^*^	0.026
RMGIC+5 wt% HAP	RMGIC (control)	24.90000^*^	<0.0001
	RMGIC+15 wt% HAP	10.46700	0.133
	RMGIC+25 wt% HAP	38.21100^*^	<0.0001
RMGIC+15 wt% HAP	RMGIC (control)	14.43300^*^	0.013
	RMGIC+5 wt% HAP	-10.46700	0.133
	RMGIC+25 wt% HAP	27.74400^*^	<0.0001
RMGIC+25 wt% HAP	RMGIC (control)	-13.31100^*^	0.026
	RMGIC+5 wt% HAP	-38.21100^*^	<0.0001
	RMGIC+15 wt% HAP	-27.74400^*^	<0.0001

In the Zirconomer group, there was a statistically significant difference in microhardness between the experimental groups (p<0.0001) (Table 5).

**Table 5:** Comparison of microhardness between the Zirconomer group HAP: hydroxyapatite

Groups	Mean	Std. deviation	P-value
Zirconomer (control)	48.71	7.67	<0.0001
Zirconomer+5 wt% HAP	76.19	7.65	
Zirconomer+15 wt% HAP	67.81	12.31	
Zirconomer+25 wt% HAP	59.30	4.44	

The 5 wt% HAP group had a microhardness of 76.19 ± 7.65, and the 15 wt% HAP group had 67.81 ± 12.31. Intragroup comparisons showed a statistically significant difference between the control group and both the 5 wt% HAP and 15 wt% HAP groups. However, no statistically significant difference was noted between the control and the 25 wt% HAP groups. Additionally, no significant differences were found between the 5 wt% HAP and 15 wt% HAP groups, or between the 15 wt% HAP and 25 wt% HAP groups (Table 6).

**Table 6:** Intragroup comparison between microhardness in the Zirconomer group HAP: hydroxyapatite *The intragroup comparison shows there is a statistically significant difference between control and 5 wt% HAP and 15 wt% HAP, there is no statistically significant difference between control and 25 wt% HAP, there is no statistically significant difference between 5 wt% HAP and 15 wt% HAP, and there is no statistically significant difference between 15 wt% HAP and 25 wt% HAP.

Groups		Mean difference (I-J)	P-value
Zirconomer (control)	Zirconomer+5 wt% HAP	-27.48100^*^	<0.0001
	Zirconomer+15 wt% HAP	-19.10500^*^	<0.0001
	Zirconomer+25 wt% HAP	-10.59700	0.050
Zirconomer+5 wt% HAP	Zirconomer (control)	27.48100^*^	<0.0001
	Zirconomer+15 wt% HAP	8.37600	0.203
	Zirconomer+25 wt% HAP	16.88400^*^	<0.0001
Zirconomer+15 wt% HAP	Zirconomer (control)	19.10500^*^	<0.0001
	Zirconomer+5 wt% HAP	-8.37600	0.203
	Zirconomer+25 wt% HAP	8.50800	0.188
Zirconomer+25 wt% HAP	Zirconomer (control)	10.59700	0.050
	Zirconomer+5 wt% HAP	-16.88400^*^	<0.0001
	Zirconomer+15 wt% HAP	-8.50800	0.188

## Discussion

When the liquid component is mixed with HAP-reinforced CGIC powder, a crucial chemical reaction occurs between the microhydroxyapatite and polycarboxylic acid. This interaction leads to the formation of a remarkably strong bond, significantly enhancing the material's overall strength [15]. The addition of microhydroxyapatite not only fortifies the bond but also results in a denser surface structure, effectively minimizing voids and contributing to the material's improved hardness. Sharafeddin et al. conducted a pivotal study in which they incorporated microhydroxyapatite into CGIC. Their findings highlighted that microhydroxyapatite-reinforced CGIC exhibited notably higher strength compared to the control group [16]. 

The mechanism behind this improvement is rooted in the ability of HAP particles to occupy the spaces between glass particles, reinforcing the overall structure and enhancing its hardness. In a related study, Sharafeddin et al. evaluated the effects of microhydroxyapatite incorporation on the microhardness of RMGIC and Zirconomer [16]. Testing concentrations of 5%, 15%, and 25% by weight, they concluded that 5 wt% incorporation yielded the highest microhardness. Lee et al. [15] also reported significant improvements in RMGIC's mechanical properties, bioactivity, and bond strength to the tooth structure with the inclusion of microhydroxyapatite.

Beyond its role in strengthening the GIC matrix, the incorporation of HAP plays a crucial role in improving the bond between the glass matrix and the core while also enhancing fluoride release. This dual function, apatite formation and fluoride release, further contributes to the superior mechanical properties of HAP-reinforced GIC. The chemical reaction between HAP and the GIC powder closely resembles the process by which GIC bonds to enamel and dentin. Polyacrylic acid in the GIC formulation displaces phosphates on the HAP surface, replacing them and allowing calcium ions to form an intermediate layer of calcium and aluminum phosphate. This creates an interface between the cement and HAP that is highly resistant to acid attack, ensuring a strong bond between the organic and inorganic components of the cement, which in turn increases its hardness [17]. Moreover, calcium in microhydroxyapatite enhances the material's microhardness, with Sharafeddin et al. confirming that 5 wt% incorporation produced the highest microhardness for both RMGIC and Zirconomer [18].

Gu et al. [7] further examined the impact of incorporating HAP/ZrO2 particles into GIC and found that this combination significantly improved the mechanical properties compared to commercial GIC formulations. However, it is important to note that an excessive amount of HAP may reduce the overall density of the set cement, potentially leading to decreased microhardness [19].

The amount of HAP added also influences the required liquid-to-powder ratio for optimal reaction. In this study, the manufacturer's recommended powder-to-liquid ratio was followed. An improper liquid ratio can compromise the material's microhardness, as voids formed from an excess of HAP can result in an inhomogeneous mixture, further reducing hardness. A similar effect was observed by researchers studying nanocrystalline calcium-deficient hydroxyapatite (nCDHA)-reinforced GIC, where increasing HAP content led to decreased microhardness. While 5 wt% and 15 wt% additions enhanced microhardness, the incorporation of 25 wt% caused a reduction in hardness [20].

Limitations

The limitation of this study is its in vitro nature, as it does not replicate the complex conditions of the oral environment, such as temperature variations, saliva interactions, and chewing forces, which could influence the material's behavior in clinical settings. Additionally, the study focused exclusively on microhardness, without considering other essential mechanical properties like compressive strength, wear resistance, and biocompatibility, which are crucial for evaluating the material's overall performance. The short-term assessment further limits the understanding of the long-term effects of incorporating microhydroxyapatite on the durability of the cements. Furthermore, while the study explored three specific concentrations of microhydroxyapatite, additional intermediate concentrations could have been investigated to identify the optimal balance between enhancing mechanical properties and maintaining material integrity. Comprehensive clinical studies and extended evaluations are necessary to confirm the potential of microhydroxyapatite-modified GICs for practical use.

## Conclusions

This in vitro study demonstrates that the incorporation of microhydroxyapatite into GICs (CGIC, RMGIC, and Zirconomer) has a significant impact on their microhardness, with lower concentrations of microhydroxyapatite consistently improving the mechanical properties of the cements. The results suggest that adding a smaller amount of microhydroxyapatite enhances microhardness without adversely affecting the structural integrity of the material. Importantly, the study highlights the potential of microhydroxyapatite to strengthen GICs, particularly at lower concentrations, which could make these materials more durable and effective in clinical applications. However, as this study focused solely on microhardness, further research is needed to assess other mechanical and biological properties, such as wear resistance, biocompatibility, and long-term performance, before microhydroxyapatite-modified GICs can be confidently recommended for widespread clinical use.

## References

[1] Abdulsamee N, Elkhadem AH (2017). Zirconomer and Zirconomer Improved (white amalgams): restorative materials for the future. Review. EC Dent Sci.

[2] Goenka S, Balu R, Sampath Kumar TS (2012). Effects of nanocrystalline calcium deficient hydroxyapatite incorporation in glass ionomer cements. J Mech Behav Biomed Mater.

[3] Upadhya PN, Kishore G (2005). Glass ionomer cement - the different generations. Trends Biomater Artif Organs.

[4] Bala O, Arisu HD, Yikilgan I, Arslan S, Gullu A (2012). Evaluation of surface roughness and hardness of different glass ionomer cements. Eur J Dent.

[5] Hershkovitz F, Cohen O, Zilberman U (2020). Microhardness of three glass-ionomer cements during setting and up to 15 days in vitro, and after 5 to 10 years in vivo. Quintessence Int.

[6] Nicholson JW (1998). Chemistry of glass-ionomer cements: a review. Biomaterials.

[7] Kheur M, Kantharia N, Lakha T, Kheur S, Al-Haj Husain N, Özcan M (2020). Evaluation of mechanical and adhesion properties of glass ionomer cement incorporating nano-sized hydroxyapatite particles. Odontology.

[8] Nicholson JW, Czarnecka B (2008). The biocompatibility of resin-modified glass-ionomer cements for dentistry. Dent Mater.

[9] Ana ID, Matsuya S, Ohta M, Ishikawa K (2003). Effects of added bioactive glass on the setting and mechanical properties of resin-modified glass ionomer cement. Biomaterials.

[10] Wilson AD (1990). Resin-modified glass-ionomer cements. Int J Prosthodont.

[11] Kobayashi M, Kon M, Miyai K, Asaoka K (2000). Strengthening of glass-ionomer cement by compounding short fibres with CaO-P2O5-SiO2-Al2O3 glass. Biomaterials.

[12] Paul S, Raina A, Kour S, Mishra S, Bansal M, Sengupta A (2020). Comparative evaluation of fluoride release and re-release and recharge potential of Zirconomer Improved and Cention. J Conserv Dent.

[13] Arita K, Yamamoto A, Shinonaga Y, Harada K, Abe Y, Nakagawa K, Sugiyama S (2011). Hydroxyapatite particle characteristics influence the enhancement of the mechanical and chemical properties of conventional restorative glass ionomer cement. Dent Mater J.

[14] Ilie N, Hickel R (2007). Mechanical behavior of glass ionomer cements as a function of loading condition and mixing procedure. Dent Mater J.

[15] Lee JJ, Lee YK, Choi BJ (2010). Physical properties of resin-reinforced glass ionomer cement modified with micro and nano-hydroxyapatite. J Nanosci Nanotechnol.

[16] Sharafeddin F, Azar MR, Feizi N, Salehi R (2017). Evaluation of surface microhardness of silver and zirconia reinforced glass-ionomers with and without microhydroxyapatite. J Dent Biomater.

[17] Sharafeddin F, Shoale S, Kowkabi M (2017). Effects of different percentages of microhydroxyapatite on microhardness of resin-modified glass-ionomer and Zirconomer. J Clin Exp Dent.

[18] Sharafeddin F, Karimi S, Jowkar Z (2019). Evaluation of the effect of micro-hydroxyapatite incorporation on the diametral tensile strength of glass ionomer cements. J Conserv Dent.

[19] Gu YW, Yap AU, Cheang P, Khor KA (2005). Effects of incorporation of HA/ZrO2 into glass ionomer cement (GIC). Biomaterials.

[20] Wan Jusoh WN, Matori KA, Mohd Zaid MH (2021). Incorporation of hydroxyapatite into glass ionomer cement (GIC) formulated based on alumino-silicate-fluoride glass ceramics from waste materials. Materials (Basel).

